# Flexitrate regional citrate anticoagulation in continuous venovenous hemodiafiltration: a retrospective analysis

**DOI:** 10.1186/s12882-019-1648-8

**Published:** 2019-12-05

**Authors:** Ilan Lenga, Wilma M. Hopman, Adam J. O’Connell, Francesca Hume, Charles C. Y. Wei

**Affiliations:** 1 Division of Nephrology, Lakeridge Health, 850 Champlain Avenue, Oshawa, Ontario L1J-8R2 Canada; 2 Lakeridge Health, Oshawa, Ontario Canada; 3 Faculty of Medicine, University of Toronto, Toronto, Ontario Canada; 4 Faculty of Medicine, Queen’s University, Kingston, Ontario Canada; 5 Kingston General Health Research Institute, Kingston, Ontario Canada

**Keywords:** Dialysis, Anticoagulation, Citrate, Heparin, AKI, Intensive care

## Abstract

**Background:**

Flexitrate, an innovative regional citrate anticoagulation (RCA) protocol, was compared to traditional RCA (tRCA) and Heparin anticoagulation protocols in intensive care patients treated with continuous renal replacement therapy (CRRT).

**Methods:**

A single-center, retrospective, cohort study, was done in a 26-bed intensive care unit in a large community hospital. Eighty dialysis sessions (Flexitrate = 2852 h, tRCA = 3580 h and Heparin = 2026 h), performed in 53 patients, were evaluated for filter life, RCA control, and metabolic control.

**Results:**

In the Flexitrate cohort, 3.8% of filters clotted, compared to 16.9% with tRCA and 28.3% with Heparin (*p* < 0.001 for Flexitrate compared to either tRCA or Heparin). Filter survival was significantly improved with Flexitrate compared to tRCA (HR 0.24, *p* = 0.018) or Heparin (HR 0.14, *p* = 0.004).

Anticoagulation control was superior with Flexitrate with Patient Ionized Calcium out of target a median of 16% of the time, compared to 27% for tRCA (*p* < 0.001). Filter Ionized Calcium was out of target a median of 6.8% of the time, compared to 23% for tRCA (*p* = 0.03).

Flexitrate produced significantly less alkalosis, hypernatremia, and hypocalcemia than tRCA, and overall metabolic control was comparable to Heparin anticoagulation. The only adverse metabolic outcome with Flexitrate was increased hypomagnesemia.

**Conclusions:**

The Flexitrate protocol extended filter life, delivered more consistent anticoagulation, and provided superior metabolic control compared to a tRCA protocol. Filter life was superior to Heparin anticoagulation, with similar metabolic control. A randomized control trial comparing these protocols is recommended.

## Background

Continuous renal replacement therapy (CRRT) is widely used in intensive care settings for the treatment of acute kidney injury in critically ill patients [1]. CRRT is optimally performed with continuous anticoagulation to allow for adequate and uninterrupted treatments. Anticoagulation is generally provided using either a continuous heparin infusion or a regional citrate anticoagulation protocol (RCA) [2].

The use of RCA has been shown to have potential benefits over heparin anticoagulation in both reduced bleeding risk and extended filter life [3]. Yet, typical protocols are plagued by complexity leading to increased risk of medical error [4]. Additionally, the use of high concentration citrate solutions (2–4%) in most protocols leads to metabolic complications [5–7].

In 2006, Tolwani, et al. described the successful implementation of a low concentration (0.5%) RCA protocol which showed excellent metabolic control and filter survival [8].

More recently, computer software has enabled algorithm driven protocols to simplify the provision of RCA and reduce nursing workload, while providing improved filter survival [9, 10].

Flexitrate^1^ is an innovative CRRT RCA protocol which combines a low concentration regional citrate solution^2^ with computer algorithmic controls designed to proactively adjust anticoagulation, thereby reducing nursing workload, and sources of error. The protocol can be used for either continuous venovenous hemodiafiltration or hemofiltration.

The Flexitrate protocol was adopted at Lakeridge Health (Oshawa, Ontario, Canada) as the preferred anticoagulation modality of CRRT for a 6 month pilot from October 2016 to March 2017. This protocol replaced the previous RCA regimen which used 4% trisodium citrate and was used preferentially to Heparin anticoagulation. This paper describes a retrospective analysis of consecutive treatments from the Flexitrate pilot period compared to historical treatments provided to consecutive CRRT recipients who received either traditional RCA or Heparin in the nine months preceding the pilot.

## Methods

The study protocol was reviewed and approved by the Research Ethics Board of Lakeridge Health Hospital, and the need for informed consent was waived.

### Descriptions of protocols

CRRT was performed using the PrismaFlex (Software Version 7.1, Baxter Corporation, Canada) with the ST-150 Set using an AN69 dialyzer, through a 12Fr catheter introduced via a central vein. Continuous venovenous hemodiafiltration (CVVHDF) mode was used with all anticoagulation protocols. Filters were changed as needed, but at least every 72 h, as per the manufacturer’s specifications. The target CRRT dose was 20–30 ml/kg/hr.

### Flexitrate protocol

A CVVHDF circuit design was adopted using Prism0cal (sodium 140 mEq/L, magnesium 0.5 mmol/L, chloride 106 mEq/L, lactate 3 mmol/L, bicarbonate 32 mEq/L) as the dialysate solution and Prism0cal B22 (sodium 140 mEq/L, magnesium 0.75 mmol/L, chloride 119.5 mEq/L, bicarbonate 22 mEq/L) as the post-filter replacement fluid. A commercially available compounded 0.5% citrate solution was introduced pre-filter via the pre-blood pump (PBP) and dosed on a mmol of citrate per liter of blood flow basis, by the treating nephrologist. The computer algorithm then automatically, and proactively, adjusted the PBP rate to account for any changes in blood pump speed, ensuring the delivery of a consistent dose of citrate. The prescribed dose of citrate was then adjusted based on the results of periodic testing of the post-filter ionized calcium, following a standard nomogram (see Additional file 1).

To avoid exceeding the desired CRRT dose, prescribers were advised to consider lower dialysate and post-filter replacement rates to account for the increase in effluent volume to match the pre-filter citrate solution.

Calcium replacement was provided via prefilled syringes of 10% calcium chloride, delivered by the syringe pump on the PrismaFlex machine. The calcium replacement was infused either through a separate central line, a supplementary lumen of a triple-lumen dialyzer catheter, or into the venous return line of the dialysis circuit. The calcium replacement rate was predicted by the computer algorithm based on the citrate and effluent rates, as well as the patient’s hematocrit. The syringe pump rate was then automatically altered to reflect any changes in these variables. Adjustments were then made to the “percent of predicted replacement” (70–120%) based on results of blood ionized calcium testing following a standard nomogram (see Additional file 1).

A detailed description of the algorithms for the citrate prescription and calcium replacement is provided in the Additional file 2.

### Traditional regional citrate anticoagulation (tRCA) protocol

The traditional regional citrate anticoagulation (tRCA) protocol evaluated in this study utilized a 4% sodium citrate solution introduced pre-filter via the Pre-Blood Pump on the PrismaFlex machine with an initial rate of 50–90 ml/hr. Prism0cal was used for both the dialysate and post-filter replacement fluid.

A calcium replacement infusion was prepared by adding 80 mL of 10% calcium chloride in 1 L of 0.9% sodium chloride (final concentration of 54.4 mmol/L). The calcium was infused via an external intravenous pump through either a separate central line or the supplementary lumen of a triple lumen dialysis catheter at an initial rate of 20 ml/hr.

All adjustments to citrate and calcium rates were made reactively and manually in response to the results of blood ionized calcium measurements according to a standard nomogram. No attempt was made to adjust citrate or calcium rates proactively when other CRRT settings were changed.

### Heparin protocol

The Heparin protocol evaluated in this study utilized a 1:1000 heparin solution delivered either via the syringe pump on the PrismaFlex machine, or via an external intravenous pump. The CRRT circuit was primed with 5000 units of heparin, and then an initial bolus of heparin was administered. A continuous infusion was maintained and adjusted based on the results of periodic testing of blood to target an activated partial thromboplastin time (aPTT) of 55–90 s.

#### CRRT sessions

During the 6 month pilot and the preceding 9 months, a total of 53 patients received CRRT. 24 patients were initiated on the Flexitrate protocol with no cross-over to the other protocols. 14 patients were initiated on the tRCA protocol with 1 patient crossing over to the Heparin protocol. 15 patients were initiated with the Heparin protocol with cross-over of 1 patient to the Flexitrate protocol and 4 patients to the tRCA protocol.

Baseline patient data were collected including demographics, diagnosis, clinical parameters, blood chemistry, blood gas results, and anticoagulation indices.

The patients received a total of 80 sessions of CRRT during this period. CRRT sessions were defined as a complete episode of CRRT treatment on a single set of orders utilizing a single anticoagulation protocol. Of these 80 sessions, 37 were performed with the Flexitrate protocol, 23 with the tRCA protocol, and 20 with the Heparin protocol. A total of 2852 h of dialysis were performed with the Flexitrate protocol, 3580 h with the tRCA protocol, and 2026 h with the Heparin protocol.

CRRT machine data were collected for each CRRT session to determine precise start/stop times, and rates for blood flow, dialysate, syringe pump, pre & post filter replacement, and fluid removal. Any concurrent use of heparin anticoagulation for an indication other than CRRT (e.g. acute coronary syndrome, deep venous thrombosis) was noted for the Flexitrate and tRCA protocols, as this could conceivably provide additional advantage to filter survival.

A total of 221 filters were used across these 80 CRRT sessions with 79 filters used in the Flexitrate, 89 in the tRCA, and 53 in the Heparin protocol sessions. Filter loss due to clotting and all filter changes were tracked.

#### Monitoring of therapy

Serum electrolytes, calcium, magnesium, and phosphate were measured twice daily for all protocols. For the Flexitrate and tRCA protocols, systemic and post-filter ionized calcium were measured just prior to and 1 h after initiation of CRRT, and then followed a protocol, being measured every 1–6 h depending on the results and if adjustments were made to citrate or calcium settings. For the Heparin protocol, activated partial thromboplastin time (aPTT) was measured just prior to and 4 h after initiation and then followed a protocol, measured every 4–12 h, depending on results or if adjustments were made to the heparin dose.

#### Statistical analyses

Data were collected in an Excel file and imported into IBM SPSS (Version 24.0 for Windows, Armonk, New York, 2017) for statistical analysis. Continuous data were tested for normality using the Shapiro Wilk method. Categorical data are presented as frequencies and percentages, and continuous data are presented as means with standard deviation, or medians with interquartile ranges (IQR), as appropriate. Baseline data and CRRT variables were compared with Pearson chi-square, Fisher exact test, independent samples t-tests or One-way ANOVA tests as appropriate. Filter survival was compared using Kaplan Meier estimates and the log rank test. Hazard ratios were estimated with 95% confidence intervals and *p*-values using Cox proportional hazard model with robust standard errors based on the sandwich variance estimate to account for recurrent events per patient. Lab parameters were compared using the Kruskal-Wallis test, and when significant, direct comparisons between groups were done using the Dunn’s Test. The Mann-Whitney U was used when it was appropriate to compare only two protocols. *P* < 0.05 was considered statistically significant, and no additional adjustments for multiple comparisons were made beyond those provided in the context of the one-way ANOVA (Tukey’s) and the Kruskal-Wallis (Dunn’s) tests.

## Results

### Clinical characteristics at initiation of CRRT

The baseline characteristics for the 53 patients are shown in Table 1. Group assignment was based on the protocol used for the patient’s first session of CRRT. At the initiation of dialysis all 3 groups were similar with respect to sepsis and urine output. No significant differences were found in age, weight, or gender.

**Table 1:** Baseline demographics

Variables	Flexitrate *N* = 24	tRCA *N* = 14	Heparin *N* = 15
Age (years)	65 ± 13	68 ± 9	68 ± 13
Weight (kg)	102 ± 31	91 ± 24	97 ± 34
Male	63%	50%	47%
Sepsis	67%	86%	60%
24 h Urine	91 ± 231 mL	77 ± 117 mL	21 ± 33 mL
Hemoglobin (g/L)	84 (74, 93)	94 (76, 103)	90 (82, 112)
Platelets	151 (76, 255)	182 (75, 235)	207 (132, 251)
Potassium (mmol/L)	5.0 (4.4, 5.5)	4.4 (3.7, 5.2)	4.6 (4.3, 5.5)
Serum CO_2_	19 (17, 23)	20 (17, 24)	24 (19, 27)
INR	1.4 (1.3, 2.0)	1.4 (1.2, 2.0)	1.2 (1.1, 1.6)
aPTT (sec)	46 (37, 55)	39 (34, 50)	42 (28, 51)

Results presented as Proportion, Mean ± SD, or Median (25th, 75th percentile). tRCA, Traditional Regional Anticoagulation

### CRRT session parameters

The CRRT parameters for the three groups are summarized in Table 2. The differences in PBP, dialysate, and replacement rates were expected as functions of the Flexitrate protocol design. However, the overall delivered dose of CRRT was significantly higher in the Flexitrate protocol (*p* = 0.01), as the reduced dialysate and replacement rates did not completely account for the increased pre-filter citrate rate. The blood flow rate and fluid removal rates did not differ between the protocols.

**Table 2:** Continuous renal replacement therapy metrics

Variables	Flexitrate *N* = 37	tRCA *N* = 23	Heparin *N* = 20	*P*-Value^*a*^
Hours of CRRT Per Session	70 (27, 90)	95 (40, 155)	88 (37, 146)	NS
Blood Flow Rate (ml/min)	150	150	150	NS
Post-filter Replacement rate (ml/hr)	741 (600, 1000)	900 (500, 1280)	1083 (589, 1443)	NS
Dialysate rate (ml/hr)	1000 (1000, 1500)	1400 (1000, 1500)	1500 (1212, 1952)	*P* < 0.001
PBP rate (ml/hr)	1496 (1373, 1504)	142 (118, 167)	144 (100, 225)	*P* < 0.001
Fluid removal rate (ml/hr)	170 ± 92	219 ± 107	186 ± 63	NS
Total effluent rate (ml/hr)	3399 ± 599	2429 ± 631	2781 ± 819	*P* < 0.001
CRRT Dose (ml/kg/hr)	33 ± 6	28 ± 7	30 ± 8	*P* = 0.01
Concurrent treatment with Heparin	5.4%	39.1%	N/A	*P* = 0.002

Results presented as Mean ± SD or Median (25th, 75th percentile). tRCA, Traditional Regional Anticoagulation. CRRT, Continuous Renal Replacement Therapy

^a^P-value calculated using ANOVA, Chi-Square, or Kruskal-Wallis tests as appropriate. No significant differences were found between the tRCA and Heparin protocols in post-hoc analysis

Significantly more sessions in the tRCA group than the Flexitrate group occurred with intravenous heparin infusions being given concurrently for another indication (39.1 vs. 5.4%).

### Filter survival

Rates of filter loss due to clotting were compared among the three protocols. For the Flexitrate protocol, only 3.8% of filters clotted, compared to 16.9% in the tRCA protocol and 28.3% in the Heparin protocol (*p* < 0.001 for Flexitrate compared to either tRCA or Heparin; No significant difference between tRCA and Heparin). Filter survival was significantly improved with the Flexitrate protocol compared to the tRCA protocol (Hazard Ratio 0.24, *p* = 0.018) and the Heparin Protocol (HR 0.14, *p* = 0.004). The tRCA protocol performed better than the Heparin protocol but this did not reach statistical significance (HR 0.59, *p* = 0.297). The predicted filter life by the Kaplan-Meier analysis, censored for filter clotting, was 79.8 ± 2.0 h for the Flexitrate protocol, as compared to 62.0 ± 2.6 h with tRCA and 60.0 ± 4.6 h with Heparin.

Given the clinical practice of pre-emptively changing filters when clotting is anticipated, a second analysis was conducted where any non-protocol filter change was treated as an event. In this analysis Flexitrate continued to show a significant advantage over the other two protocols (*p* = 0.014). Filters lasted longer on the Flexitrate protocol compared to the tRCA protocol (HR 0.41, *p* = 0.013), with no difference found between tRCA and Heparin protocols (HR 0.96, *p* = 0.932).

**Fig. 1:**
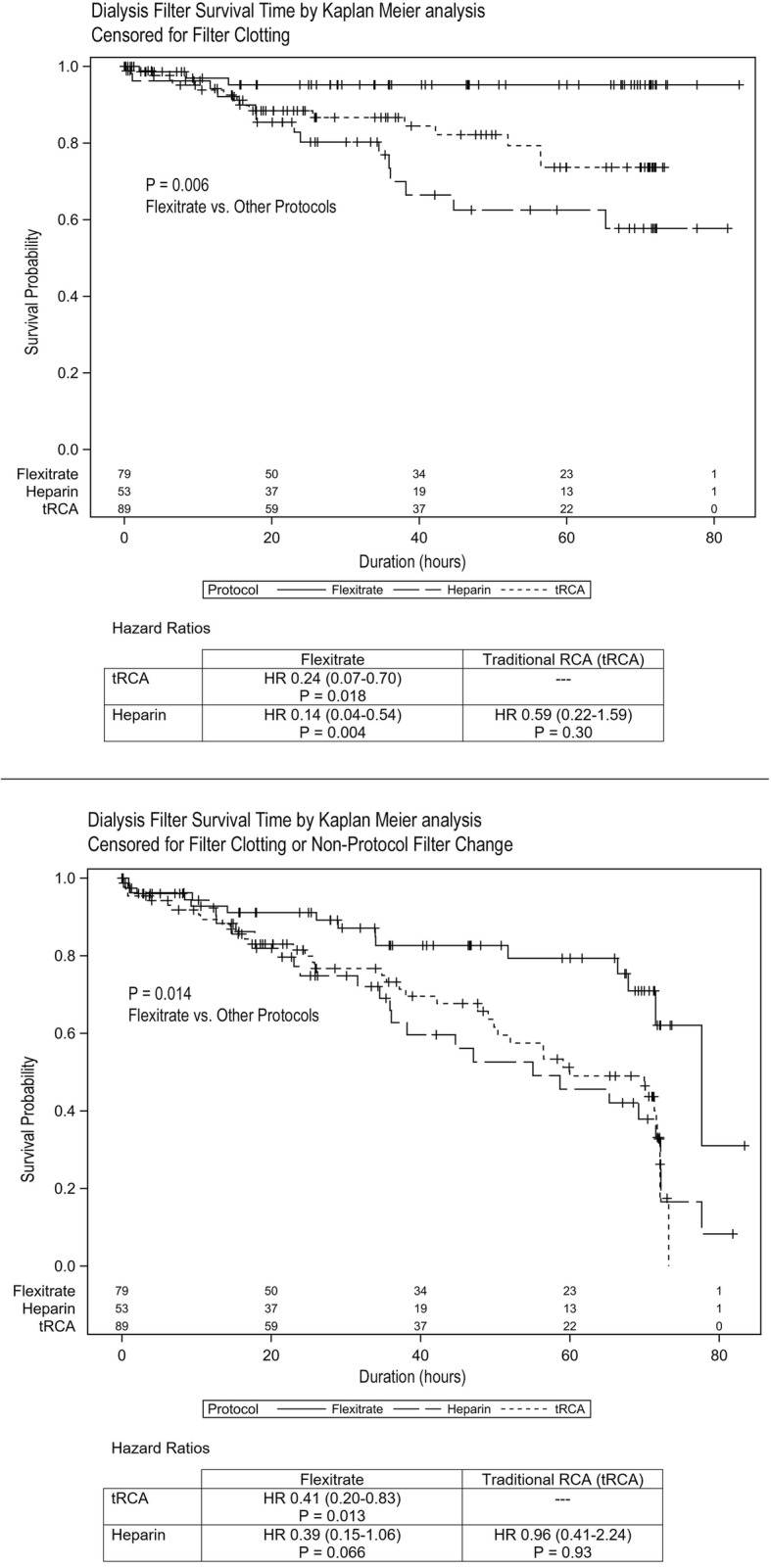
Dialysis Filter Survival Time by Kaplan Meier Analysis

Kaplan-Meier Filter survival curves for both analyses are presented in Fig. 1.

### Regional citrate anticoagulation control

**Fig. 2:**
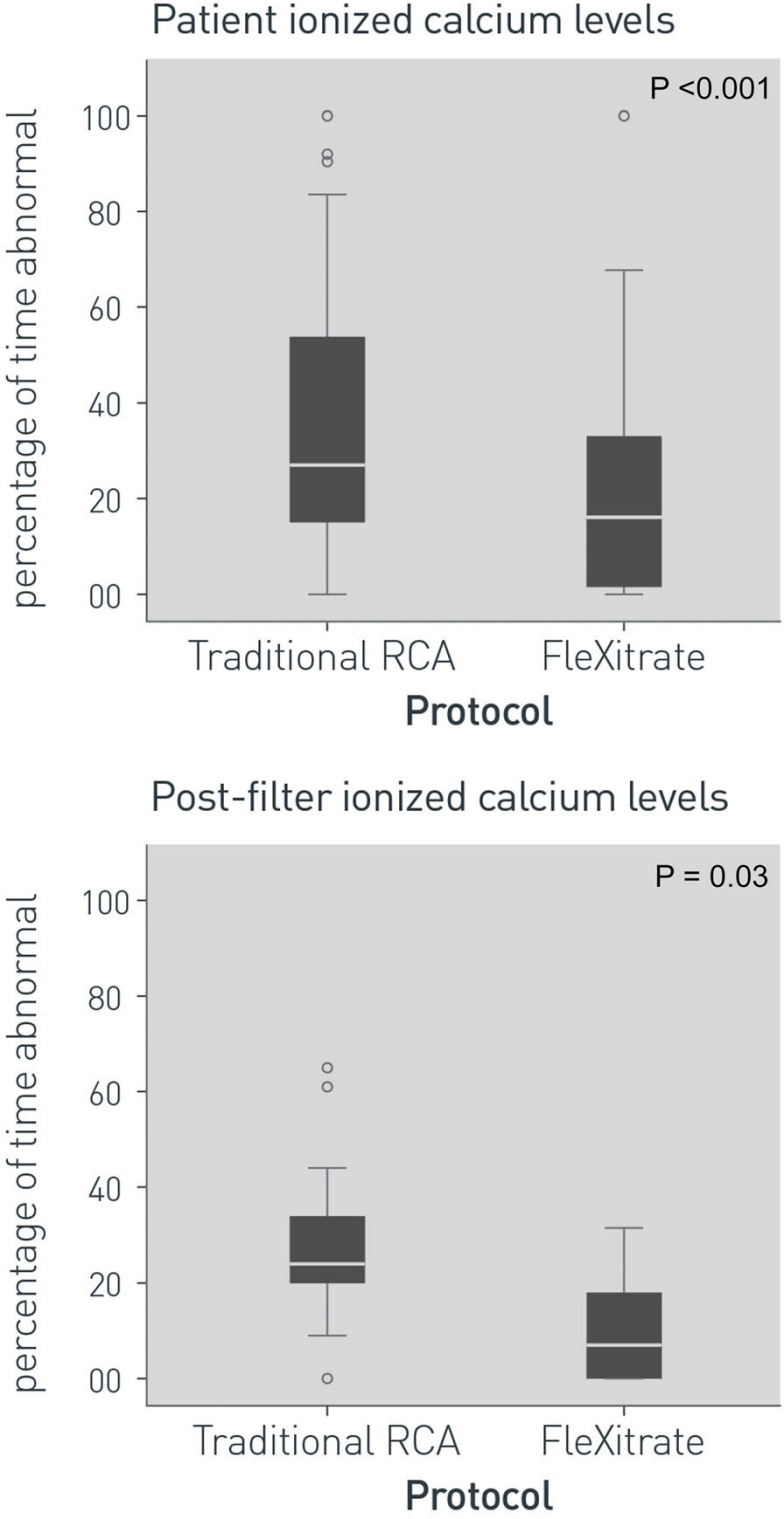
Anticoagulation control on Continuous Renal Replacement for Flexitrate and Traditional Regional Citrate Anticoagulation

Comparisons of the amount of time Post-filter ionized calcium and Patient ionized calcium were outside of target, are presented in Fig. 2. The Flexitrate protocol significantly outperformed the tRCA protocol. Consequently, a median of only 4.8 (IQR 3.9–6.3) tests of Post-Filter ionized calcium per day were required under this protocol, compared with 7.6 (IQR 5.7–8.8) tests per day required with the tRCA protocol (*P* = 0.001).

### Metabolic control

**Fig. 3:**
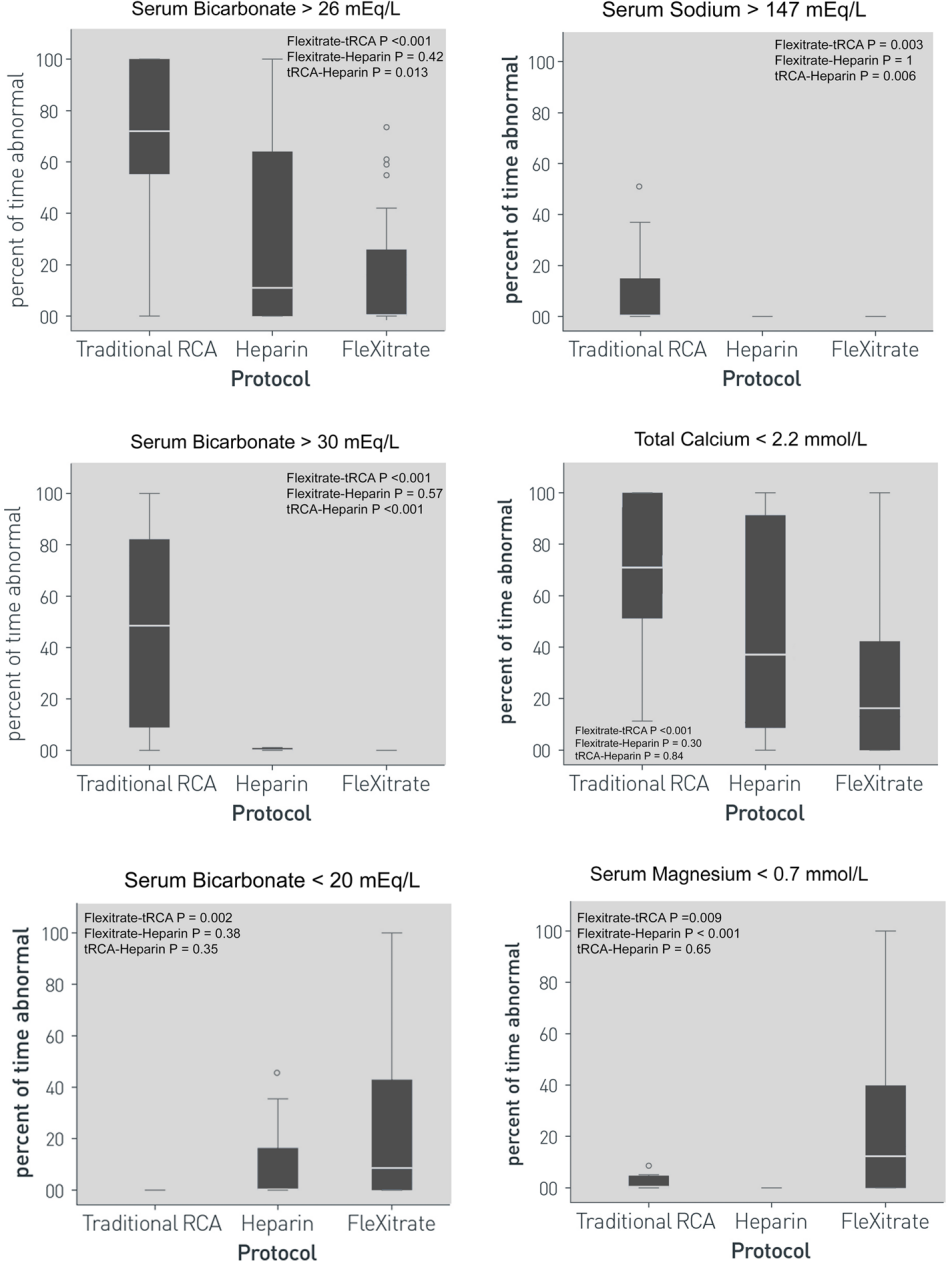
Metabolic control on Continuous Renal Replacement for Flexitrate, Traditional Regional Citrate Anticoagulation, and Heparin protocols

Comparisons of metabolic control parameters are presented in Fig. 3. Alkalosis was significantly reduced using the Flexitrate protocol with less time where serum bicarbonate levels exceeded 26 mmol/L and 30 mmol/L. High levels of alkalosis in the tRCA group, masked underlying acidosis, which was statistically similar in both the Flexitrate and Heparin groups but more frequent than in the tRCA group.

Hypernatremia was uncommon, but unique to the tRCA group. Hyponatremia was exceedingly rare in all the protocols, and no statistically significant difference was found between protocols. Hypocalcemia was more frequent in the tRCA protocol, present 72% of the time compared to 16% in Flexitrate and 37% in the Heparin protocol. Citrate toxicity, defined as the presence of hypercalcemia, with a ratio of 2.5X between total and ionized levels of calcium, was very rare with Flexitrate and tRCA with no significant difference found.

Hypomagnesemia (defined as Mg level less than 0.7 mmol/L) occurred more frequently during Flexitrate sessions, present for 13% of the time, as compared to 1.6% in the tRCA group and 0% in the Heparin group.

No significant differences were found for potassium, anion gap, chloride, phosphate, arterial and venous pH, hemoglobin, or platelets.

## Discussion

The Flexitrate protocol provided a significant filter survival advantage over both other protocols. This held true both for overt clotting of filters and for all non-protocol filter changes. Filters were 4.2 times more likely to clot with the tRCA protocol, and 7.1 times more likely to clot with the Heparin protocol, compared to the Flexitrate protocol.

The fact that more patients in the tRCA group than the Flexitrate group were receiving concurrent treatment with intravenous Heparin (39.1 vs. 5.4%) risked confounding the results, by providing a filter survival advantage to the tRCA group. Despite this, Flexitrate still outperformed the tRCA protocol, strengthening confidence in the result.

In this analysis, the tRCA protocol did not show a statistically significant filter survival advantage over Heparin (HR 0.59, *p* = 0.147). Although some studies have suggested that such an advantage exists [11], our results are consistent with a subsequent metanalysis [2]. This suggests that it is the unique properties of the Flexitrate protocol that enhances filter survival. This advantage may be due to the additional pre-filter blood dilution provided by using a 0.5% citrate solution, or the superior control of citrate anticoagulation parameters as shown in this study.

Both filter ionized calcium and patient ionized calcium levels remained in target more often with the Flexitrate protocol, providing more consistent anticoagulation than the tRCA protocol. Consequently, a median of 2.8 fewer tests of filter ionized calcium were required per day, with the corollary of reduced workload and lab costs.

Regional citrate protocols are associated with metabolic disturbances in acid-base, sodium, and calcium homeostasis. In this study the Flexitrate protocol produced less alkalosis, hypernatremia, and hypocalcemia than the tRCA protocol, and overall metabolic control was comparable to Heparin anticoagulation. The only unique adverse metabolic outcome in the Flexitrate protocol was increased hypomagnesemia. Presumably this is due to the larger volume of pre-filter dilution with magnesium free citrate solution in the Flexitrate protocol. This occurred despite a dialysate solution in the Flexitrate protocol that was higher in magnesium (0.75 mmol/L) than in the tRCA protocol (0.5 mmol/L) with identical post-filter replacement fluids.

This study has the limitations of an observational, single centre, retrospective analysis. Since the cohorts in the study are from different time periods, co-intervention bias could have been introduced. No changes in protocols or clinical practice occurred during the period of the study. The difference in concurrent treatment with intravenous heparin for non-CRRT indications, discussed above, was due to differences in patient comorbidity in these cohorts and not attributed to a change in CRRT practice patterns. Although no changes in co-interventions are known to have occurred, the possibility of this bias remains a limitation of the study.

The complete switchover from tRCA to Flexitrate for the period analyzed does eliminate selection bias in those comparisons, but selection bias between Flexitrate and Heparin or between tRCA and Heparin groups could exist. Also, not all CRRT and filter sessions were strictly independent as multiple sessions occurred in the same patient. Care was taken to employ conservative statistical methods in the analysis of filter sessions to account for this clustering.

## Conclusions

In this observational, single centre, retrospective analysis, the Flexitrate regional citrate anticoagulation protocol significantly extended filter life, delivered more consistent anticoagulation, and provided superior metabolic control compared to a traditional regional citrate anticoagulation protocol. Filter life was also superior to heparin anticoagulation, while providing similar metabolic control. A randomized control trial comparing these protocols is recommended.

## Supplementary information

**Additional file 1.** Nomograms used for adjusting citrate dose and calcium replacement in the Flexitrate protocol.

**Additional file 2.** Detailed description of the computerized algorithms used by the Flexitrate protocol.

## Data Availability

The datasets used and/or analysed during the current study are available from the corresponding author on reasonable request.

## Abbreviations

aPTT: activated partial thromboplastin time
CRRT: Continuous renal replacement therapy
CVVHDF: Continuous venovenous hemodiafiltration
HR: Hazard ratio
IQR: Inter quartile range
RCA: Regional citrate anticoagulation
tRCA: traditional regional citrate anticoagulation

## Footnotes

1 “FleXitrate” refers to a therapeutic concept that allows integrated Prismaflex management, via the citrate-calcium module, of both low concentration citrate and calcium and is not a product name for any approved drug or product in Canada. It is also the term used to describe the low concentration citrate protocol used in this paper. FleXitrate is a trademark of Baxter International Inc. or its subsidiaries.

2 This study was conducted within Canada where there are currently no commercially available citrate products approved by Health Canada for use in CRRT. The use of citrate for CRRT in Canada is off-label and is considered investigational. The low concentration (0.5%) citrate solution used in this study is compounded as ordered by the physician, using commercially available tri-sodium citrate (4% citrate) solutions.
