# Supplementary material for: Flexitrate regional citrate anticoagulation in continuous venovenous hemodiafiltration: a retrospective analysis
Source: BMC Nephrol. 2019 Dec 5;20:452. doi: 10.1186/s12882-019-1648-8 (PMC6896713; doi:10.1186/s12882-019-1648-8)
Supplement: Supplementary file 1 — Additional file 1. Nomograms used for adjusting citrate dose and calcium replacement in the Flexitrate protocol. [file 12882_2019_1648_MOESM1_ESM.docx]

# Nomogram used for adjusting Citrate Dose

| Post-Filter Ionized Calcium | Target Citrate Concentration Change | Repeat Post-Filter Ionized Calcium |
| --- | --- | --- |
| Less than 0.15 mmol/L | Decrease by 0.3 mmol/L and check the system for set-up flaws | in 1 hour |
| 0.15 – 0.19 mmol/L | Decrease by 0.2 mmol/L | In 1 hour |
| 0.20 – 0.24 mmol/L | Decrease by 0.1 mmol/L | In 1 hour |
| 0.25 - 0.39 mmol/L | No change |  |
| 0.40 - 0.45 mmol/L | No change |  |
| 0.46 - 0.50 mmol/L | Increase by 0.1 mmol/L | in 1 hour |
| 0.51 - 0.55 mmol/L | Increase by 0.2 mmol/L | in 1 hour |
| Greater than 0.55 mmol/L | Increase by 0.3 mmol/L and check the system for set-up flaws | in 1 hour |

# Nomogram used for adjusting Calcium Replacement

| Patient’s Ionized Calcium | Calcium Chloride  Bolus and Rate Change | Repeat Ionized Calcium |
| --- | --- | --- |
| Less than 0.75 mmol/L | Bolus 3 g calcium chloride in 100 mL 0.9% Sodium Chloride over 2 hours and increase calcium compensation by 30% | 1 hour after calcium bolus completed |
| 0.76 - 0.85 mmol/L | Bolus 2 g calcium chloride in 100 mL 0.9% Sodium Chloride over 1 hour and increase calcium compensation by 20% | 1 hour after calcium bolus completed |
| 0.86 - 0.90 mmol/L | Bolus 1 g calcium chloride in 100 mL 0.9% Sodium Chloride over 1 hour and increase calcium compensation by 10% | 1 hour after calcium bolus completed |
| 0.91 – 0.99 mmol/L | - If post-filter ionized calcium below target then no change  - If post-filter ionized calcium at or above target then increase calcium compensation by 5% | in 1 hour |
| 1 - 1.35 mmol/L | No change |  |
| 1.36 – 1.45 mmol/L | Decrease calcium compensation by 5% | in 1 hour |
| Greater than  1.45 mmol/L | Decrease calcium compensation by 10% | In 1 hour |
