# Supplementary material for: Flexitrate regional citrate anticoagulation in continuous venovenous hemodiafiltration: a retrospective analysis
Source: BMC Nephrol. 2019 Dec 5;20:452. doi: 10.1186/s12882-019-1648-8 (PMC6896713; doi:10.1186/s12882-019-1648-8)
Supplement: Supplementary file 2 — Additional file 2. Detailed description of the computerized algorithms used by the Flexitrate protocol. [file 12882_2019_1648_MOESM2_ESM.docx]

# Flexitrate Algorithms

Excerpted from: Prismaflex Operator Manual version 7.xx, Chapter 8: Anticoagulation Methods

## Citrate Prescription

In citrate anticoagulation, PBP ﬂow rate is kept proportional to blood ﬂow rate and computed by the software through the equation:

Q_pbp_ = (Qb × D_cit_) / [Cit]

Where Q_pbp_ is PBP ﬂow rate (ml/h), Qb is Blood ﬂow rate (ml/h), D_cit_ is Citrate dose expressed in millimole per liter of blood (mmol/l blood) and [Cit] is citrate concentration of the PBP solution (mmol/l).

Citrate dose is deﬁned as the amount of citrate infused per liter of patient's blood treated, expressed in mmol/l blood. Citrate dose is the user-controllable setting.  Citrate concentration is the sum of citrate and citric acid concentration as deﬁned in Service mode for the selected citrate solution.

Blood ﬂow rate affects the PBP citrate ﬂow rate. A change of the blood ﬂow rate will automatically result in:

• Change of the PBP citrate ﬂow rate.

• Change of the treatment dose (ml/kg/hr)

• Change of the estimated patient citrate load

## Calcium Prescription

In “Citrate – Calcium, Prismaﬂex syringe pump” anticoagulation method, the syringe ﬂow rate is kept proportional to the estimated calcium loss rate in efﬂuent. It is computed by the software of the Prismaﬂex system through the equation:

Q_syr_ = CaComp × J_Ca_ / [Ca] - Q_rep_ × [Ca_rep_] / [Ca]

Where CaComp is the calcium compensation, Q_syr_ is syringe ﬂow rate (ml/h), J_Ca_ is estimated calcium loss rate in efﬂuent (mmol/h), [Ca] is calcium concentration of the syringe solution (mmol/l), Q_rep_ is the replacement ﬂow rate(ml/h), and [Ca_rep_] is calcium concentration of the replacement solution in post-dilution (mmol/l).

Calcium compensation is deﬁned as the relative dosage of calcium infusion to compensate for the estimated calcium loss in efﬂuent, expressed in percentage. Calcium compensation is the user-controllable setting. Calcium concentration is the concentration of the calcium solution as deﬁned in Service mode for the selected calcium solution.

Estimated calcium loss represents the amount of calcium removed in the efﬂuent. Its calculation is based on several factors:

• the estimation of calcium clearance as a function of blood, PBP, replacement, dialysate and patient ﬂuid removal rates, as well as on disposable set in use and patient's hematocrit

• the assumption that the patient Total Calcium is 2.2 mmol/l

• the assumption that pre-dilution replacement solutions and/or dialysate solutions are calcium-free.

Any change of one or more ﬂow rates or of anticoagulation settings affects the calcium syringe ﬂow rate, reﬂecting the estimated calcium clearance change and the inﬂow of calcium from replacement solutions used in post-dilution.

Note: in a similar way, any update of the patient’s hematocrit in the Modify Settings screen during the treatment affects the calcium syringe ﬂow rate.
